# Photoluminescence emission and Raman enhancement in TERS: an experimental and analytic revisiting

**DOI:** 10.1515/nanoph-2023-0882

**Published:** 2024-02-02

**Authors:** Yu-Ting Chen, Quan Liu, Felix Schneider, Marc Brecht, Alfred J. Meixner, Dai Zhang

**Affiliations:** Institute of Physical and Theoretical Chemistry, Eberhard Karls University of Tübingen, 72076 Tübingen, Germany; Process Analysis and Technology (PA&T), Reutlingen University, 72762 Reutlingen, Germany

**Keywords:** TERS, plasmonics, gap-mode, gap distance, cobalt phthalocyanine, copper phthalocyanine

## Abstract

An analytic model is used to calculate the Raman and fluorescence enhancement of a molecule in between two closely spaced gold nanospheres. Instead of using the conventional approach that only the dipolar plasmonic mode is considered, we calculate the electric field enhancement in the nanometre sized gap, by taking account of the higher order modes in one gold sphere, which couples to the dipolar mode of the other sphere. The experimental confirmation is performed by gap-dependent tip-enhanced Raman spectroscopy (TERS) measurements. The photoluminescence and Raman enhancement are both observed with different growing trends as the gap width decreases. Red-shift of the background spectra is observed and implies the increasing coupling between the nanospheres. This analytic model is shown to be able to interpret the enhancement mechanisms underlying gap-dependent TERS experimental results.

## 1 Introduction

As a combination of advanced scanning probe technique and optical spectroscopy, tip-enhanced Raman spectroscopy (TERS) offers high spatial resolutions and high chemical sensitivity. Since the first demonstration on inverted optical microscopes with transparent samples [1], [2], [3], [4], TERS has been extended to non-transparent substrates using refractive side-illumination [5], [6], [7] and parabolic mirrors (PM) [8], [9], [10]. Furthermore, the available commercialized TERS apparatuses enable researchers of broad scientific backgrounds access to this highly demanding technique. In all TERS configurations, a plasmonic metal tip plays a particularly important role because of its ability to confine and enhance the electromagnetic field of incident excitation and radiation in the near field [11], [12], [13], [14], [15]. Although the recent works in the context of quantum plasmonics [16], [17], [18], [19], [20], [21] have introduced new understandings of the signal enhancement at extremely small tip–sample distance (*d* < 1 nm), plasmon mode induced electromagnetic field enhancement is still dominant for the general TERS measurements.

Widely accepted electromagnetic enhancements of Raman scattering have been analysed theoretically and evaluated experimentally for silver nanoaggregates [22], [23]. Several numerical studies have explored the enhancement for different tip geometries, tip materials and tip–sample distances as well as the excitation polarization [24], [25], [26], [27], [28], [29], [30], [31], [32], [33], [34], [35], [36], [37]. The enhancement for small tip–sample distance *d* has been found to follow a *d* ^−10^ dependence [38]. A well-known phenomenon in company with the plasmonic near field enhancement is the presence of a broad background in the TERS spectra [38], [39], [40]. Different mechanisms have been suggested for its origin, such as surface plasmon-induced photoluminescence (PL) [41], [42], [43], [44]. Several experimental studies [38], [45] have investigated the influence of the tip–sample distance on the enhancement of both the PL background and Raman signal, where the enhancement of both signals has either been treated in the same manner as the field enhancement [46] or only been described using a phenomenological model [45]. The connections between the PL background and Raman signal enhancement have been also discussed in [39], [40], [47]. The evolution of plasmon-induced PL has been found to be strongly correlated with the Raman modes. Though numerical simulations addressing the fourth power of electric field enhancement offer a satisfactory description of the enhancement for small tip–sample gaps [40], they need to be further considered to explain the different behaviours of PL and Raman modes when the gap is larger (>10 nm) [38], [46]. Recent extensive experimental and theoretical discussions concerning the emission from plasmonic nanostructures [48], [49], [50], [51], [52], [53], [54], [55] have deepened the understanding on the origin of plasmon-induced PL. Although it is still under debate whether hot carriers induced ‘PL’ [53], [55] or electronic Raman scattering [51], [52] should be considered as responsible for the observed PL background, both pictures confirm the importance of an enhanced electromagnetic field in the vicinity of nanostructures. Therefore, it is important to further explore the tip–sample distance dependence of TERS spectra to reveal the field enhancement [56], [57]. In order to elucidate the relation of PL background and TERS spectra and to gain further insight into the enhancement mechanism, it is worth to revisit the correlated behaviour of Raman and PL background intensities at varying tip–sample distances.

In this work, we collected TERS spectra from copper and cobalt phthalocyanine thin films deposited on smooth gold samples for different tip–sample distances, and an analytical model [58], [59], [60], [61] is used to interpret the enhancement of PL and Raman spectra. The tip–sample configuration is considered as a coupled nanosphere dimer [62], [63], [64], where the tip is approximated as a small nanosphere and the substrate as a much larger sphere. The gap dependences are properly captured by considering the different origin of PL and Raman. A good agreement of the simulation with our experimental results validates the applied analytical model.

## 2 Experimental

### 2.1 Sample preparation

Two types of samples were prepared for the different experimental approaches. The first sample (referred as Sample 1) is a cobalt phthalocyanine (CoPc, nominal thickness: 2 nm) film deposited on an Au substrate by organic molecular beam deposition under ultrahigh vacuum conditions (UHV). The Au substrate is prepared by evaporating a 50 nm Au layer on Si-substrate with 2 nm chromium as the adhesion layer between the Au and the Si.

The second sample (referred as Sample 2) is a monolayer copper phthalocyanine (CuPc, nominal thickness: 0.3 nm) film thermally evaporated under UHV on Au (111) single crystal.

### 2.2 Optical microscope setup

**Figure 1:**
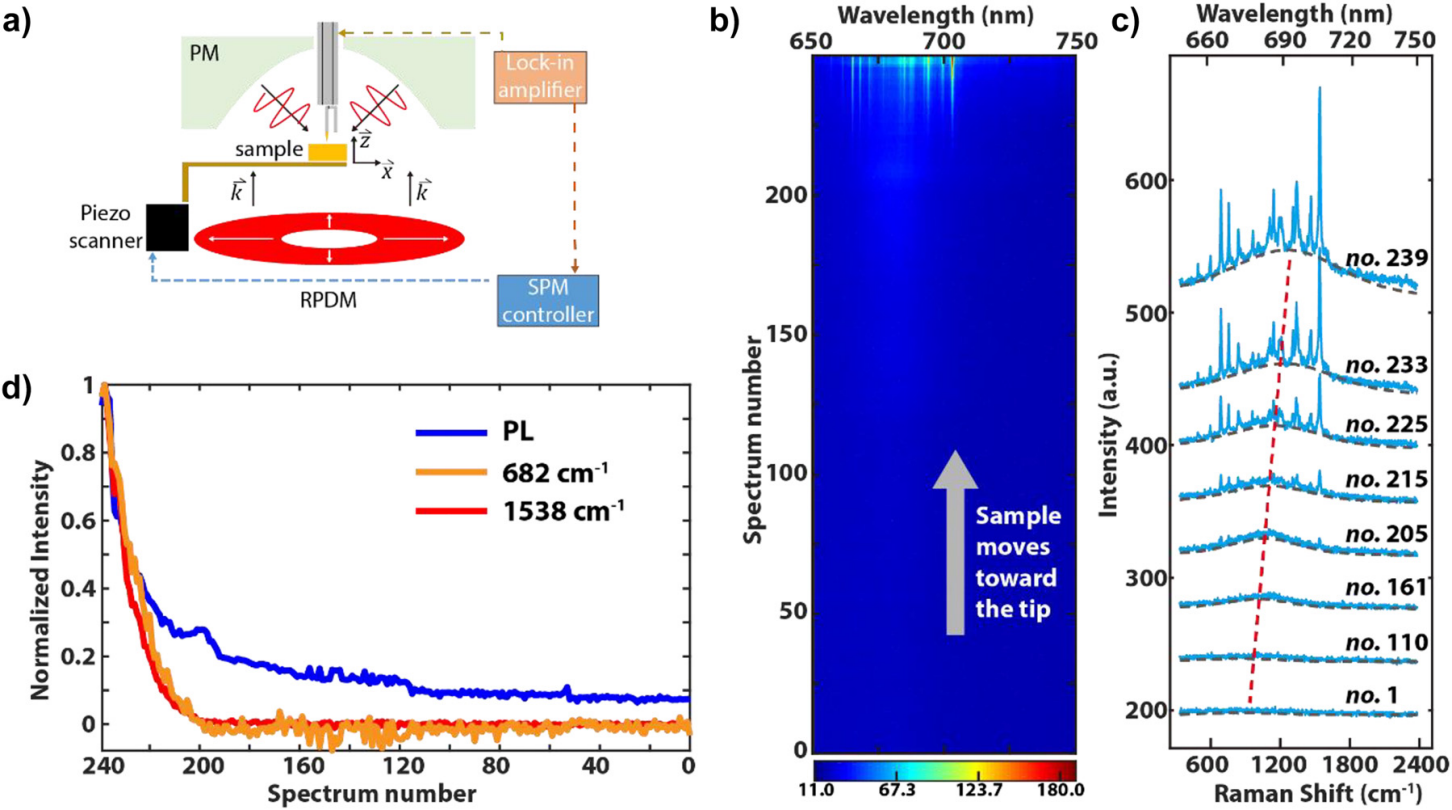
Raman and PL intensities measured versus tip-sample distance. (a) Schematic drawing of the experimental setup. The sample is mounted on a sample holder and its position with respect to the tip is controlled via a shear-force feedback. An Au tip is glued on one prone of the quartz tuning fork. A radially polarized laser beam (RPDM) is focused by the PM onto the sample. To collect an optical image, the sample is raster scanned through the focus via a piezo scanner. All the electronic control is made through a SPM controller. (b) Optical emission spectra with the sample moving towards the Au tip stepwise. The *y*-axis indicates the number of spectra, which is the largest at the shortest tip–sample distance (as indicated by the grey arrow). The *x*-axis is the wavelength. The colour bar indicates the spectral intensity, with red as the highest value. (c) A set (from number 1 to number 239) of gap-dependent TERS spectra (blue) that are fitted with Voigt function (grey dashed line). The red dashed line is a guide for the eye for the shift of PL peak maxima while approaching the sample to the tip. (d) Normalized intensities of two Raman modes (682, 1538 cm^−1^) and PL while approaching the sample to the tip (from spectral number 1 to 240).

The layout of the whole optical microscope is described elsewhere [65]. As shown in Figure 1(a), a PM is used to illuminate the sharp gold tip by a diffraction limited focal field and at the same time to collect the scattered and luminescence photons from the tip and sample. The excitation laser beam is converted into a radially polarized doughnut mode (RPDM) through a mode converter [66]. The sample position is controlled by a XYZ piezo scanner (P-517.3CL, Physik Instrumente). The scanning probe Au tip is prepared by electrochemically etching gold wires [67] and is rigidly mounted on a quartz tuning fork. The Au tip approaches to the diffraction limited laser focus from above into the PM. To centre the Au tip at the laser focus, its position is fine-adjusted to ensure a minimal and symmetric Rayleigh scattering and corresponding PL patterns of the tip. The sample moves towards the optical focus with shear piezo stacks on the sample holder. The quartz tuning fork is mechanically dithered by a piezo-tube at the resonance frequency (32 kHz), and the pre-amplified output signal of the tuning fork goes to a lock-in amplifier (Ametek 7270 DSP). The demodulated phase shift signal is sent to the SPM controller (RHK Technology, SPM100) for the feedback control of the tip–sample gap distance [68]. At large tip–sample distances (>10 nm), the sample approach is performed without feedback control.

For optical detection, the TERS signal passes through a beam splitter and two notch filters (central wavelength 633 nm, StopLine^®^ single-notch filter) and is recorded by a single-photon counting avalanche photodiode (APD, SPCM-AQR-14, Perkin Elmer, MA, USA), as well as by a liquid-nitrogen cooled CCD camera coupled with a spectrometer (Acton Research, SpectraPro 300i, Perkin Elmer, MA, USA). Sample 1 was measured without a feedback control using the excitation laser of *λ*_*ex*_ = 636 nm. During the approach that was started at a rather large tip–sample distance, each spectrum was acquired with an integration time of 10 s while the sample approached stepwise to the Au tip. Sample 2 was measured by varying the tip–sample distance within a range of 30 nm. The wavelength of the excitation laser is *λ*_*ex*_ = 632.8 nm. The smallest tip–sample distance S is maintained constant and is estimated around 4 ± 1 nm. This sample position is, therefore, assigned as the position of *L* = 0 nm (see inset of Figure 3(a)). The sample moved towards the tip from *L* = 0 nm to *L* = 30 nm at a constant rate of 0.6 nm/s, while the spectra were collected with the integration time of 1 s simultaneously.

## 3 Results and discussion

The experimental layout is presented in Figure 1(a). To maximize the plasmon excitation along the tip axis and hence field enhancement at the tip apex, we use RPDM excitation to provide a large longitudinal electric field component ${\overset{⇀}{E}}_{z}$ in the focus [69], [70], [71]. Figure 1(b) shows 240 continuously measured TERS spectra with an integration time of 10 s per spectrum while the sample was approaching to the tip stepwise without feedback control until the tip crashed onto the sample. The spectra from 1 to 239 were taken at decreasing tip–sample distances. Since at the beginning of the tip approach, the sample position was very far away from the tip, no Raman signal is visible until the last 40 spectra. As has been well reported, the TERS effect starts to be visible at tip–sample gap distances of less than 10 nm [38], [45]. Therefore, we estimate the beginning sample position where the spectrum 1 is taken to be about 60 nm away from the tip. The PL background and TERS signal were separated by fitting Voigt profiles to raw TERS spectra. Since the quantum yield (QY) of intrinsic PL from CuPc and CoPc is generally very weak [72], [73], [74], the broad continuum spectral background is very unlikely from the molecular fluorescence emission. As shown in Figure 1(c), with the decreasing tip–sample distances, the maximum of the spectral background shifts to longer wavelengths (highlighted by the red dash line), this behaviour was assigned to the increasing coupling between the tip and substrate for gap widths <5 nm [45]. The gap-distance dependent spectral intensities (normalized to one) of the PL and two different Raman peaks at 682 and 1538 cm^−1^ are plotted in Figure 1(d). Both Raman intensity and PL intensity are increasing as the sample is approaching the Au tip. Notably, the *x*-axis starts with the spectrum no. 240 that corresponds to the smallest gap distance. The intensity of spectrum 1 is mostly due to the PL from the Au tip since the sample is far away from the focus at that point. The PL intensity appears to be rather constant for spectra 1 to about 100 and increases slowly afterwards, as shown by the blue curve. Differently, the Raman intensities (orange and red curves) start to increase only after spectrum 200 and show a steeper slope than the PL signal.

**Figure 2:**
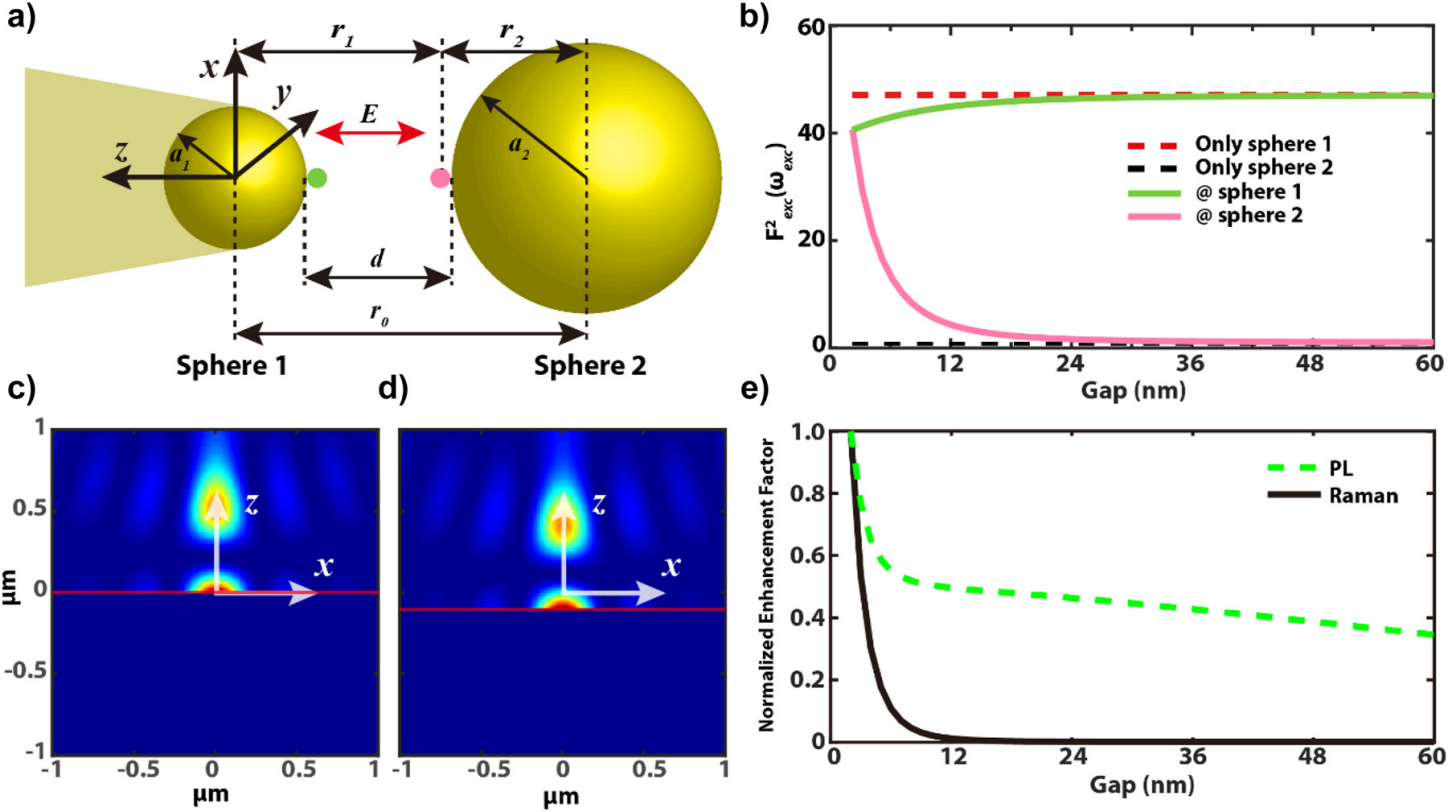
Simulated gap-distance dependent PL and Raman enhancement factors. (a) Diagram of two closely placed gold nanospheres 1 and 2. Their centres define the *z*-axis of a Cartesian coordinate system, and the centre of sphere 1 defines the origin. (b) Simulated enhancement of the excitation field intensity at a distance of 1 nm away (green dot in (a)) from sphere 1 with (solid green) and without (dashed red) the presence of sphere 2 as a function of the gap *d*. Same results at a distance of 1 nm away (pink dot in (a)) from sphere 2 with (solid pink) and without (dashed black) the presence of sphere 1. (c)–(d) Simulated intensity of the longitudinal ( ${\overset{⇀}{E}}_{z}$ ) component of the excitation field above a gold sample surface in the focal region of a parabolic mirror (NA = 1) created with a radially polarized laser beam (*λ*_*ex*_ = 632.8 nm). The small sphere is always located in the focus of the parabolic mirror at *z*_*tip*_ = 0 nm, and the tip–sample gap *d* is varied by approaching the sample from below towards the tip. In (c) and (d), *Z*_*sample*_ is at 0 nm and −100 nm below the focus, respectively. (e) Normalized enhancement factor of PL (green dash line) and Raman (black solid line) for varying gap *d*.

In the next section, we apply an often used classical, analytic model to describe the enhanced field in the tip–sample gap [59], [75] and interpret the gap-distance dependent PL and Raman intensities shown in Figure 1. In this model, the electrostatic problem is solved first to describe the inhomogeneous field distribution in the gap, and in the second step, the time evolution is included. The TERS configuration is approximated as two closely placed gold nanospheres with rotational symmetry about the *z*-axis defined by the centres of the two spheres, i.e. the optical axis as shown in Figure 2(a), with an incident focal field *E*_*foc*_ oriented in the *z*-direction. The gold tip is treated as a small sphere (sphere 1) with radius *a*_1_ = 15 nm (according to the SEM image) centred in the focus of the PM and the substrate as a large sphere (sphere 2) with radius *a*_2_ = 1000 nm. The inhomogeneous field distribution created by the opposite surface charges mirrored between the two spheres in the presence of the large sphere is modelled by spherical harmonics [59], [75], [76]. The interaction between the two spheres is considered as the coupling of the bare plasmonic modes from the two counterparts, as it has been considered for calculating the field enhancement in the nanoparticle-on-mirror system configuration [64]. The surrounding is air. The centre-to-centre distance between the two spheres is *r*_0_, and *d* is the gap between the two surfaces. We limit *d* ≥ 2 nm in our simulations in accordance with our shear force experiments; hence, nonlocal effects and quantum tunnelling that appear for narrower gaps are not considered [19], [77]. The polarization of the electric field is oriented along the *z*-axis.

The rate equation of the relative amplitude of the *l*th order mode $A_{l}=\sqrt{\frac{1}{4}\varepsilon_{0}\varepsilon_{D}V_{\text{eff,l}}}E_{\max,l}$ for this configuration is given in [75]:

(1) $$\begin{array}{cc} \frac{dA_{1}^{\left(m\right)}}{dt} & =i\left(\omega -\omega_{1}\right)A_{1}^{\left(m\right)}-i\overset{\infty }{\underset{l=1}{\sum }}\omega_{1l}\kappa_{1l}^{\left(mn\right)}A_{l}^{\left(n\right)}\\ & \;-\frac{1}{2}\gamma_{1}^{\left(m\right)}A_{1}^{\left(m\right)}+K_{in}^{\left(m\right)}\\ \frac{dA_{l}^{\left(m\right)}}{dt} & =i\left(\omega -\omega_{l}\right)A_{l}^{\left(m\right)}-i\omega_{1l}\kappa_{1l}^{\left(nm\right)}A_{1}^{\left(n\right)}\\ & \;-\frac{1}{2}\gamma_{l}^{\left(m\right)}A_{l}^{\left(m\right)};\;l\geq 2 \end{array}$$

Here, *V*_eff*,l*_ is the effective volume of the *l*th mode and *E*_max,*l*_ is the maximum field strength at the surface of the gold sphere, *ω* is the angular frequency of the incident laser radiation and the superscripts *m*, *n* = 1 or 2 stand for the spheres 1 and 2. *ω*_*l*_ and *γ*_*l*_ are the resonance frequency and the damping constant of the *l*th mode, $\omega_{1l}=\sqrt{\omega_{1}\omega_{l}}$ is the reduced frequency, and $K_{in}^{\left(m\right)}$ determines the laser power coupled into the nanosphere [78]. $\kappa_{1l}^{\left(mn\right)}$ is the coupling coefficient between the dipole mode in one sphere and all the other modes in the other sphere [75]. The total enhanced electric field *E*_*gap*_ in the gap at the position of the pink dot as defined by *r*_1_ and *r*_2_ (see Figure 2(a)) is the vector sum of the incident field and the field created by the polarized spheres, $E_{\mathrm{gap}}=E_{\mathrm{foc}}+E\left(r_{1}\right)$ . The latter can be solved for steady state conditions of equation (1),

(2) $$\begin{array}{ll} E\left(r_{1}\right) & =E_{\max,1}^{\left(1\right)}{\left(\frac{a_{1}}{r_{1}}\right)}^{3}+E_{\max,1}^{\left(1\right)}\overset{\infty }{\underset{l=2}{\sum }}\frac{\omega_{1l}\kappa_{1l}^{\left(mn\right)}}{\left(\omega_{l}-\omega \right)+i\frac{\gamma }{2}}\\ & \;\times \frac{l+1}{2}{\left(\frac{a_{1}}{a_{2}}\right)}^{\frac{3}{2}}{\left(\frac{a_{2}}{r_{2}}\right)}^{l+2}+E_{\max,1}^{\left(2\right)}{\left(\frac{a_{2}}{r_{2}}\right)}^{3}\\ & \;+E_{\max,1}^{\left(2\right)}\overset{\infty }{\underset{l=2}{\sum }}\frac{\omega_{1l}\kappa_{1l}^{\left(mn\right)}}{\left(\omega -\omega_{l}\right)+i\frac{\gamma }{2}}\frac{l+1}{2}{\left(\frac{a_{2}}{a_{1}}\right)}^{\frac{3}{2}}\\ & \;\times {\left(\frac{a_{1}}{r_{1}}\right)}^{l+2} \end{array}$$

The field enhancement factor is defined as the ratio of the gap-field *E*_*gap*_ to the incident focal field *E*_*foc*_ and can be written as

(3) $$F_{\mathrm{field}}\left(r_{1}\right)=\left|\frac{E\left(r_{1}\right)}{E_{\mathrm{foc}}}\right|+1$$

The origin of plasmon-related broad spectral backgrounds appearing in enhanced Raman spectra have been explained with different theoretical models, including direct plasmon radiation [44], [50], [79], Purcell effect enhanced hot carrier recombination [53] and inelastic light scattering [52]. However, it is consistent in all the models to describe the PL enhancement *F*_*PL*_ as a combination of excitation enhancement and emission enhancement, which results to $F_{PL}\propto F_{\mathrm{exc}}^{2}\left(\omega_{\mathrm{exc}}\right)\cdot V_{\mathrm{pla}}\left(\omega_{\mathrm{exc}}\right)\cdot F_{\mathrm{emi}}^{2}\left(\omega_{\mathrm{emi}}\right)$ . *F*_*exc*_ and *F*_*emi*_ are the excitation field enhancement and emission field enhancement, respectively. *V*_*pla*_ is the volume of the sphere that is accessed by the plasmonic gap field. Due to the localization property of plasmon modes, we can calculate the field enhancement only in the vicinity of both spheres and multiply it by a constant *V*_*pla*_ to evaluate the PL enhancement *F*_*PL*_. As for the Raman enhancement *F*_*TERS*_, the molecules are only located on the sample substrate; hence, it can be evaluated by the field enhancement close to the sphere 2 as $F_{\mathrm{TERS}}\propto F_{\mathrm{exc}}^{2}\left(\omega_{\mathrm{exc}}\right)\cdot F_{\mathrm{emi}}^{2}\left(\omega_{\mathrm{emi}}\right)$ .

To evaluate the PL and Raman enhancement, we treated equation (3) numerically. We should consider that the excitation laser frequency is higher than the PL and Raman frequency. Therefore, we need to calculate the enhancement factor for the incident field and emission field separately. The excitation laser frequency is scaled as *ω*_*exc*_ = 1.08 ⋅ *ω*_*emi*_ = 1.08 ⋅ *ω*_1_ to be consistent with the experimental condition, and the gap varies from 60 nm to 2 nm. Figure 2(b) shows the simulation results of $F_{\mathrm{exc}}^{2}\left(\omega_{\mathrm{exc}}\right)$ at two positions marked by the green dot and the pink dot that are 1 nm away from the surfaces of sphere 1 and sphere 2. The field enhancement at 1 nm away from a single sphere is also shown as dashed lines for comparison. As there is no gap for single spheres, the respective enhancement factors are constant. Additionally, the larger sphere 2 possesses a larger radiation dipole and most of the energy dissipates via radiation, while the smaller sphere 1 focuses the field in its vicinity. Therefore, sphere 1 produces a larger field enhancement than sphere 2. For gaps with *d* ≥ 20 nm, coupling between the two spheres is weak; thus, the enhancement is then similar to the situation of the single sphere. For gaps with *d* < 20 nm, increased coupling between the two spheres leads to the higher enhancement in the vicinity of sphere 2 and a lower one close to the sphere 1. The enhancement at the gap centre finally reaches a $F_{\mathrm{exc}}^{2}\left(\omega_{\mathrm{exc}}\right)=41$ at *d* = 2 nm. A similar behaviour is obtained for $F_{\mathrm{emi}}^{2}\left(\omega_{\mathrm{emi}}\right)$ (not shown here), but the enhancement factor is 209 at *d* = 2 nm, which is much larger since the emission is in resonance with the plasmon gap mode.

Before evaluating the total enhancement, we need to consider the intensity profile of a focused RPDM beam in the focal volume in the vicinity of a gold tip. The intensity profile of the longitudinal ( ${\overset{⇀}{E}}_{z}$ ) component is dominating in the focus [80] of a RPDM beam. In our measurements, the tip is always located in the focus and the substrate moves towards the tip in focus to change the gap distance. The reflection of the incident field by the substrate leads to a standing wave pattern with rotational symmetry about the optical axis, and the intensity profile along the optical axis has a clearly visible nodal zero field intensity about 250 nm above the sample surface, which needs to be considered for larger gaps especially. Figure 2(c) and (d) are calculated field intensity profiles in the *xz*-plane in the focus region of the PM (NA = 1) created with a RPDM beam (*λ*_*ex*_ = 632.8 nm) by assuming that the focus is at *z* = 0 nm (c) and the substrate (gold) is at *z* = 0 nm or *z* = −100 nm (d), respectively. We ignore the impact of the tip when calculating the far field focus field intensity profiles since the tip is small compared to the laser spot. Figure 2(e) shows the simulated PL enhancement (green dash line) and Raman enhancement (black line). The maxima are normalized to one for the comparison with the experimental results. A dramatic increase of the enhancement factor for both the PL and Raman at a gap smaller than 10 nm is nicely reproduced in our simulation. A distinct difference can be seen at *d* > 12 nm, where the enhancement of Raman vanishes and the PL intensity only decreases slightly. The PL intensity is mainly related to the electromagnetic enhancement from sphere 1, which is considered as stationary in the focus for the simulation. The PL intensity reduces slowly due to the change in the laser intensity profile, which is 35 % of its maximum value at *d* = 60 nm. The decreasing tendency is slower than the experimental results, which decreases to less than 20 % at *d* = 60 nm as shown in Figure 1(d).

**Figure 3:**
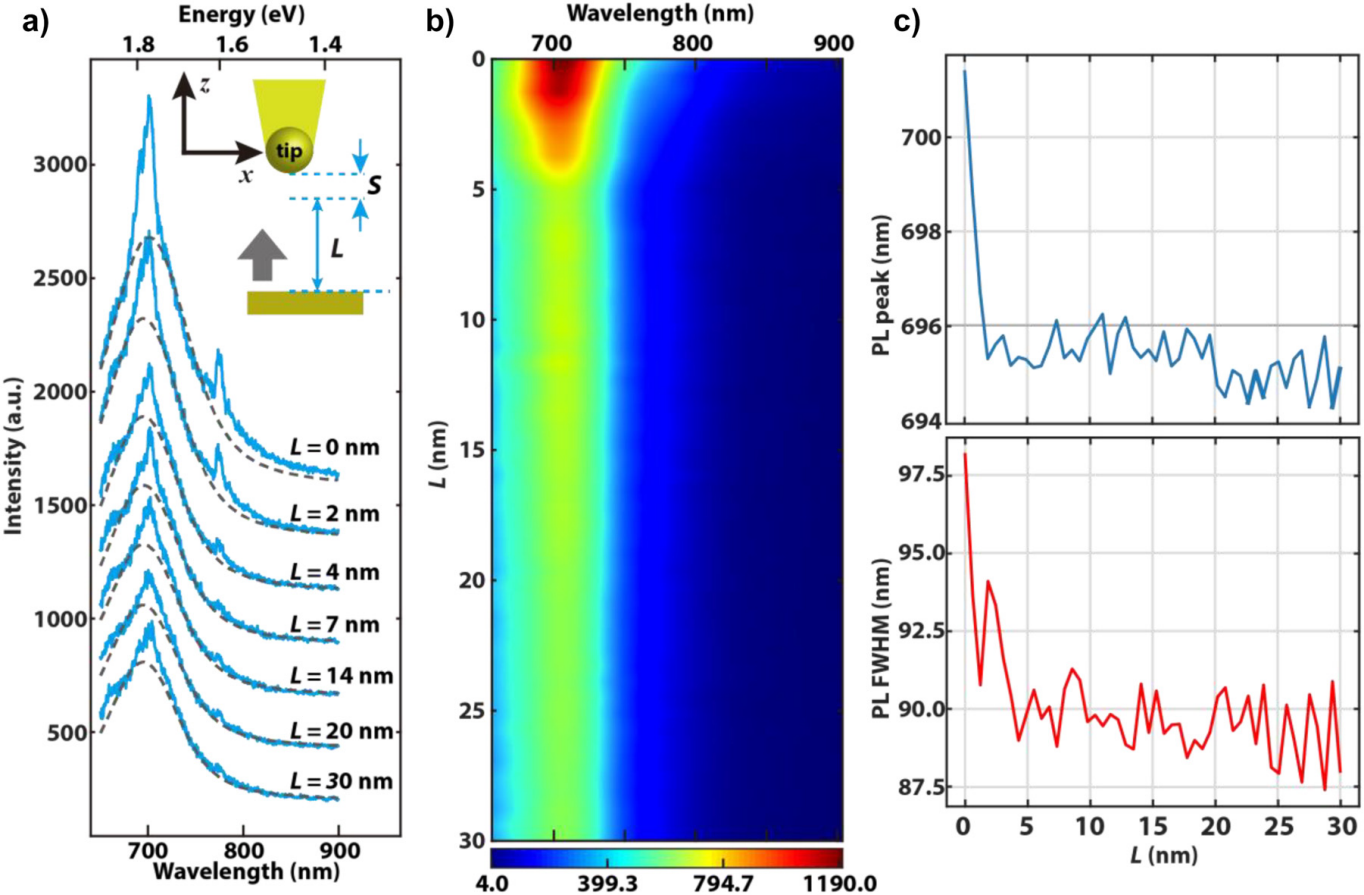
PL spectral position and FWHM measured at different tip-sample distances. (a) Representative TERS spectra (blue) for different sample shift L with Voigt fitting (grey dashed). Au tip and sample are maintained by shear-force feedback with initial gap *S*. (b) Fitted PL spectra plotted for the sample shift *L*. (c) The evolution of peak position and FWHM of PL extracted from Voigt fitting.

**Figure 4:**
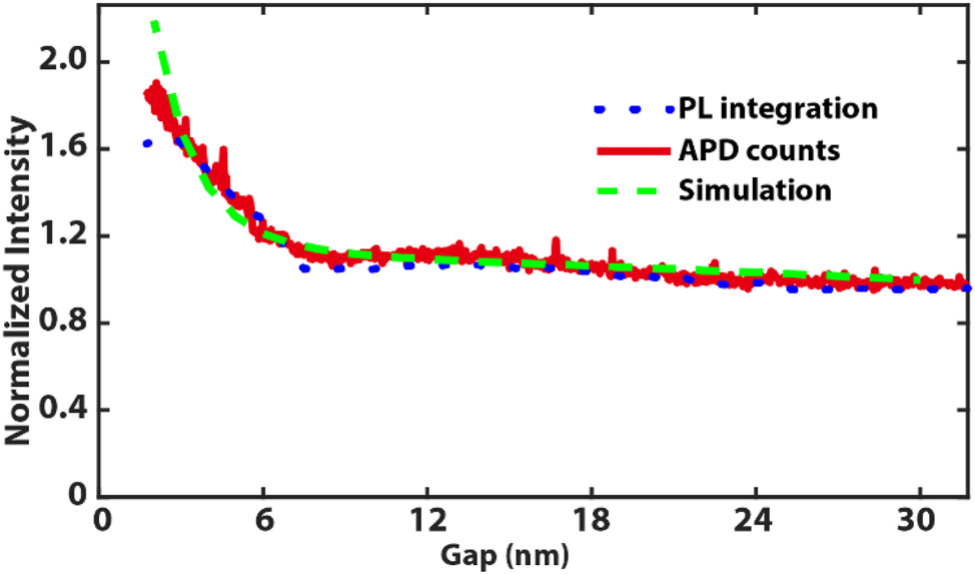
Gap-dependent PL intensity plot obtained from the experimental tip-enhanced spectra (blue) and APD (red). Green dash line is the simulation result overlaid on top.

To validate the model quantitatively, we performed PL measurements by controlling the gap distances via an accurate feedback loop. The measurement is performed on a sample with a monolayer CuPc film on a single gold crystal. This substrate has negligible surface roughness to avoid the involvement of a surface-enhanced effect. A series of spectra taken during the sample approaching towards the tip are shown in Figure 3(a). Here, we define the tip–sample distance as *d* = *L* + *S*. The definition of *L* and *S* is illustrated as the inset in Figure 3(a). In the experiment, the gap variation is limited between *L* = 0 nm and 30 nm. Hence, we can ignore any influences from the sample defocus and excitation intensity variations at large gap distances. The minimum tip–sample distance is around 2 nm (*L* = 0 nm, and *S* = 2 nm) due to the shear-force feedback loop, as show in inset of Figure 3(a). The excitation laser wavelength is 632.8 nm. The Raman peaks are not properly resolved since a 150 grooves/mm grating was used to collect the full PL spectra. The PL intensity profile is derived by fitting the TERS spectra with a Voigt function, which are plotted as grey dashed lines in Figure 3(a). Figure 3(b) shows the PL spectra collected during the sample movement towards the tip in a rate of 0.6 nm/s. In total, 50 spectra were acquired with the integration time of 1 s per spectrum. The gap-dependent PL intensities and widths are shown in Figure 3(c). The peak position is around 1.78 eV (696 nm) with a full width at half maximum (FWHM) of ∼0.25eV (92 nm), which agrees with the experimental results of [45]. Similar to the results shown in Figure 1, at the small gap distances *L* ≤ 5 nm, the PL intensity increases significantly with a broader peak form. Thanks to the precise feedback loop, we know that the maximum gap distance is 30 nm and the final gap *S* is less than 5 nm. Hence, it is possible to directly compare the simulation and experimental results. The simulated PL intensity with the gap *d* varies over 2 nm–30 nm and is plotted in Figure 4. The PL spectra intensity and APD signal (binning time 10 ms) collected in parallel against the movement of the sample is also shown. The intensity minimum is normalized to 1 for a better comparison. The simulation and experimental results are nicely overlaid after applying a 2 nm shift for experimental results, indicating the final gap *S* ≈ 2 nm. As suggested by the simulation, there is a fast increase of PL signal when the gap *d* < 10 nm. Unlike the gap-distance dependent Raman signal, PL intensity does not drop to zero since the tip is in the focus during the entire measurement. The deviation of the simulation and experiment is increasing when *d* < 4 nm. The simulation suggests an PL enhancement factor of 2.2 compared to the PL at *d* = 30 nm, while it is 1.8 for the experiment. This can be explained by increased Landau damping in a narrow gap [19], [50], which is not considered in our model.

## 4 Conclusions

An analytic model that accounts for the coupling of the tip and the substrate is adopted to explain the different tip–sample distance dependences of Raman and PL signals. Raman enhancement is simulated by the electromagnetic field enhancement around the probe molecule, while the PL enhancement simulation accounts for the field enhancement as well as the volume accessed by the plasmonic field. Different from the Raman signal that arise from the probe molecule, the origin of PL is the volume accessed by the plasmonic field. The rapid increases of Raman and PL intensities at gaps *d* < 10 nm is assigned to the stronger coupling between the tip and the substrate, leading to a large field enhancement in vicinity of the substrate. The Raman enhancement vanishes at *d* > 12 nm. However, the simulation predicts that PL enhancement only decrease slightly since the tip stays in the focus and contributes for most of the PL signal. This is verified by the measurement with precisely controlled tip–sample distance. Although the analytic model did not consider non-classical effects, such as non-local effect, Landau damping and quantum tunnelling, the nice consistency of the simulation and the experimental results proves its effectivity.
